# Supplementary material for: Synthetic inhibition of the SUMO pathway by targeting the SAE1 component via TAK-981 compound impairs growth and chemosensitizes embryonal and alveolar rhabdomyosarcoma cell lines
Source: Mol Cell Biochem. 2025 Jun 23;480(10):5501–19. doi: 10.1007/s11010-025-05336-6 (PMC12515125; doi:10.1007/s11010-025-05336-6)
Supplement: Supplementary file 1 — Supplementary file1 (PPTX 58659 KB) [file 11010_2025_5336_MOESM1_ESM.pptx]

## Slide 1
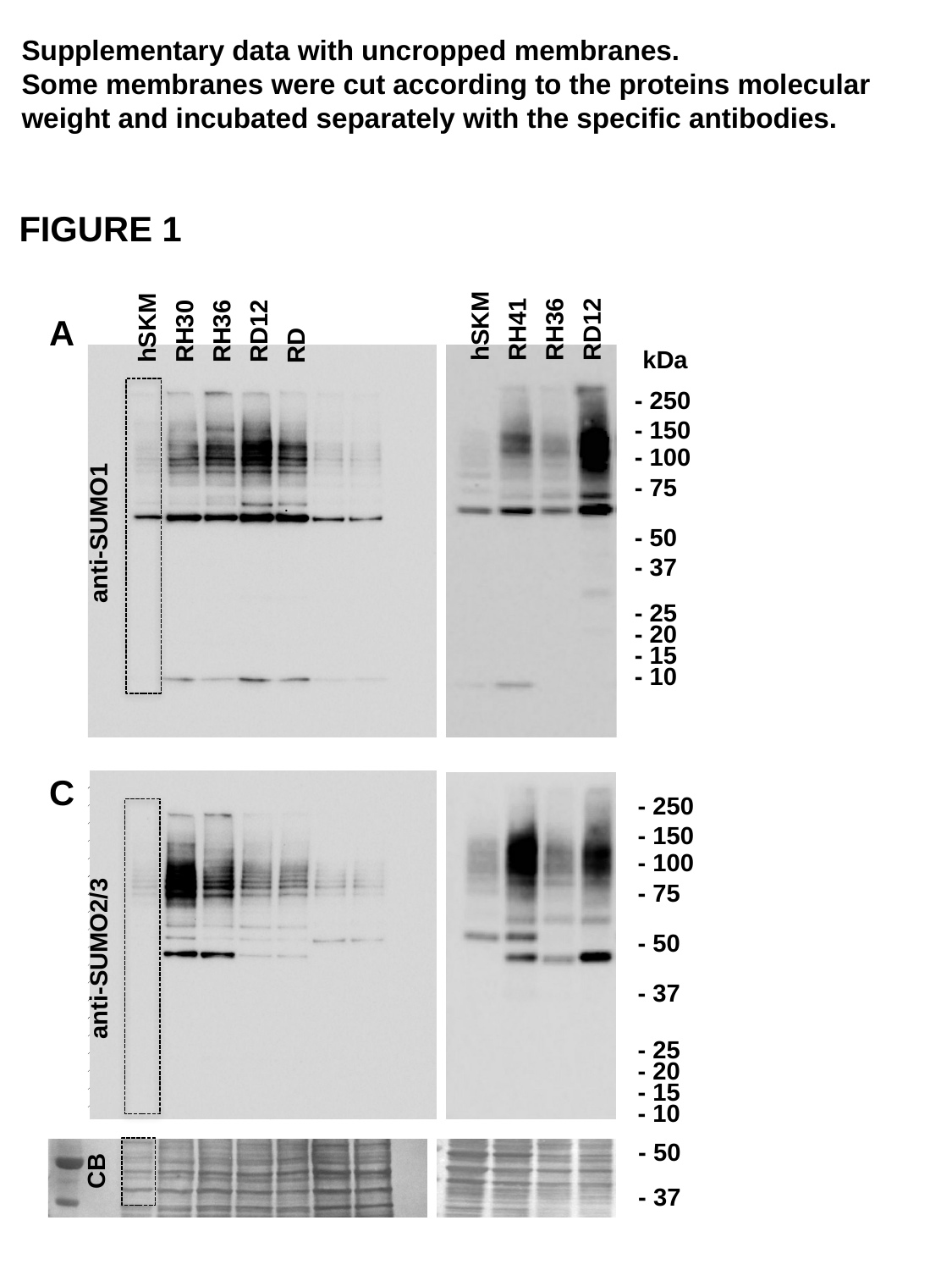

Supplementary data with uncropped membranes.
Some membranes were cut according to the proteins molecular weight and incubated separately with the specific antibodies.
FIGURE 1
RD
hSKM
RH41
RH36
RD12
hSKM
RH30
RH36
RD12
A
kDa
- 250
- 150
- 100
- 75
- 50
- 37
- 25
- 20
- 15
- 10
- 250
- 150
- 100
- 75
- 50
- 37
- 25
- 20
- 15
- 10
- 50
- 37
anti-SUMO1
C
anti-SUMO2/3
CB

## Slide 2
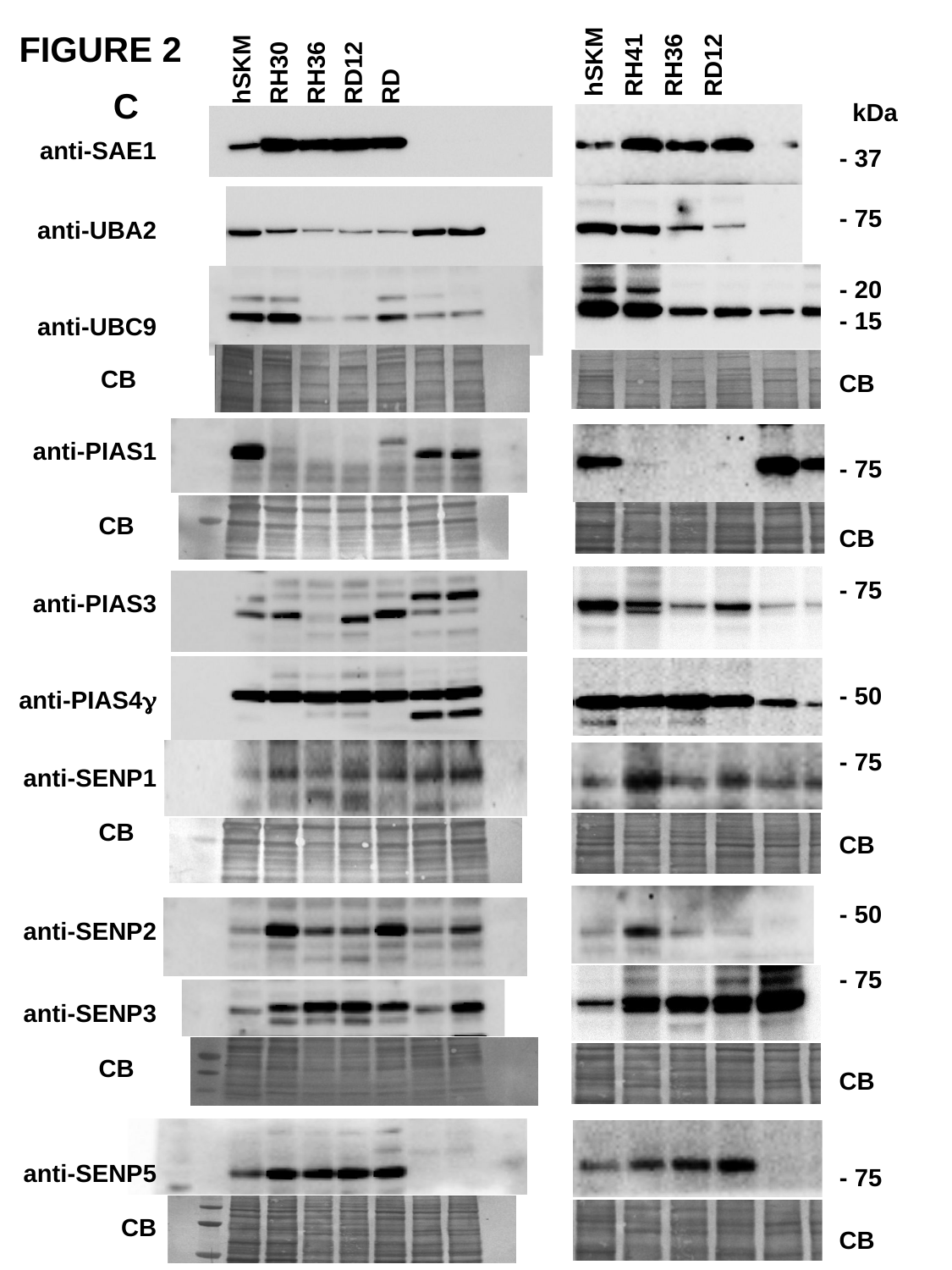

RD
hSKM
RH41
RH36
RD12
hSKM
FIGURE 2
RD12
RH30
RH36
C
kDa
anti-SAE1
- 37
- 75
anti-UBA2
- 20
- 15
anti-UBC9
CB
CB
anti-PIAS1
- 75
CB
CB
- 75
anti-PIAS3
- 50
anti-PIAS4g
- 75
anti-SENP1
CB
CB
- 50
anti-SENP2
- 75
anti-SENP3
CB
CB
anti-SENP5
- 75
CB
CB

## Slide 3
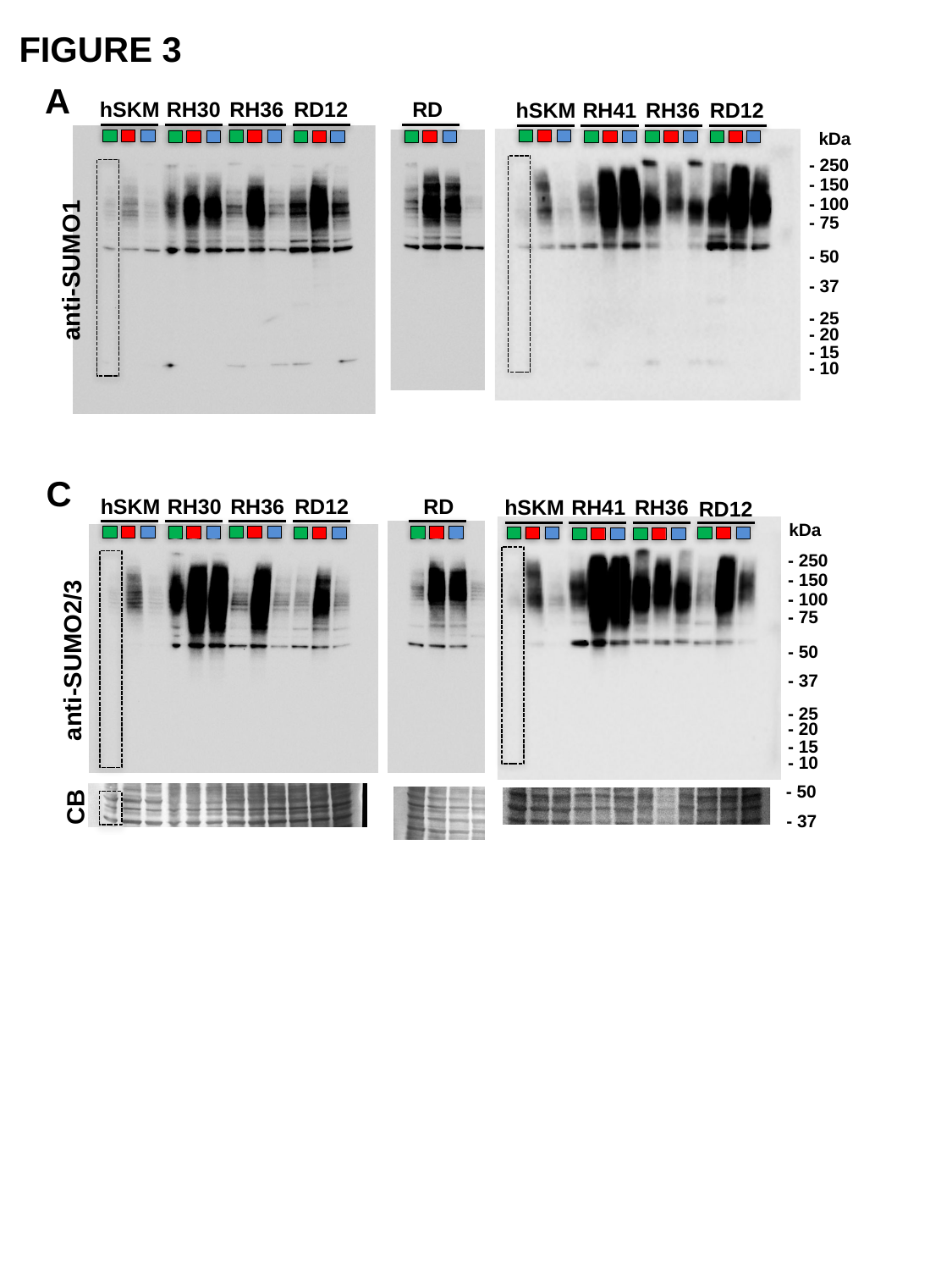

FIGURE 3
A
hSKM
RH30
RH36
RD12
RD
hSKM
RH41
RH36
RD12
kDa
- 250
- 150
- 100
- 75
- 50
- 37
- 25
- 20
- 15
- 10
anti-SUMO1
C
hSKM
RH30
RH36
RD12
RD
hSKM
RH41
RH36
RD12
kDa
- 250
- 150
- 100
- 75
- 50
- 37
- 25
- 20
- 15
- 10
anti-SUMO2/3
- 50
CB
- 37

## Slide 4
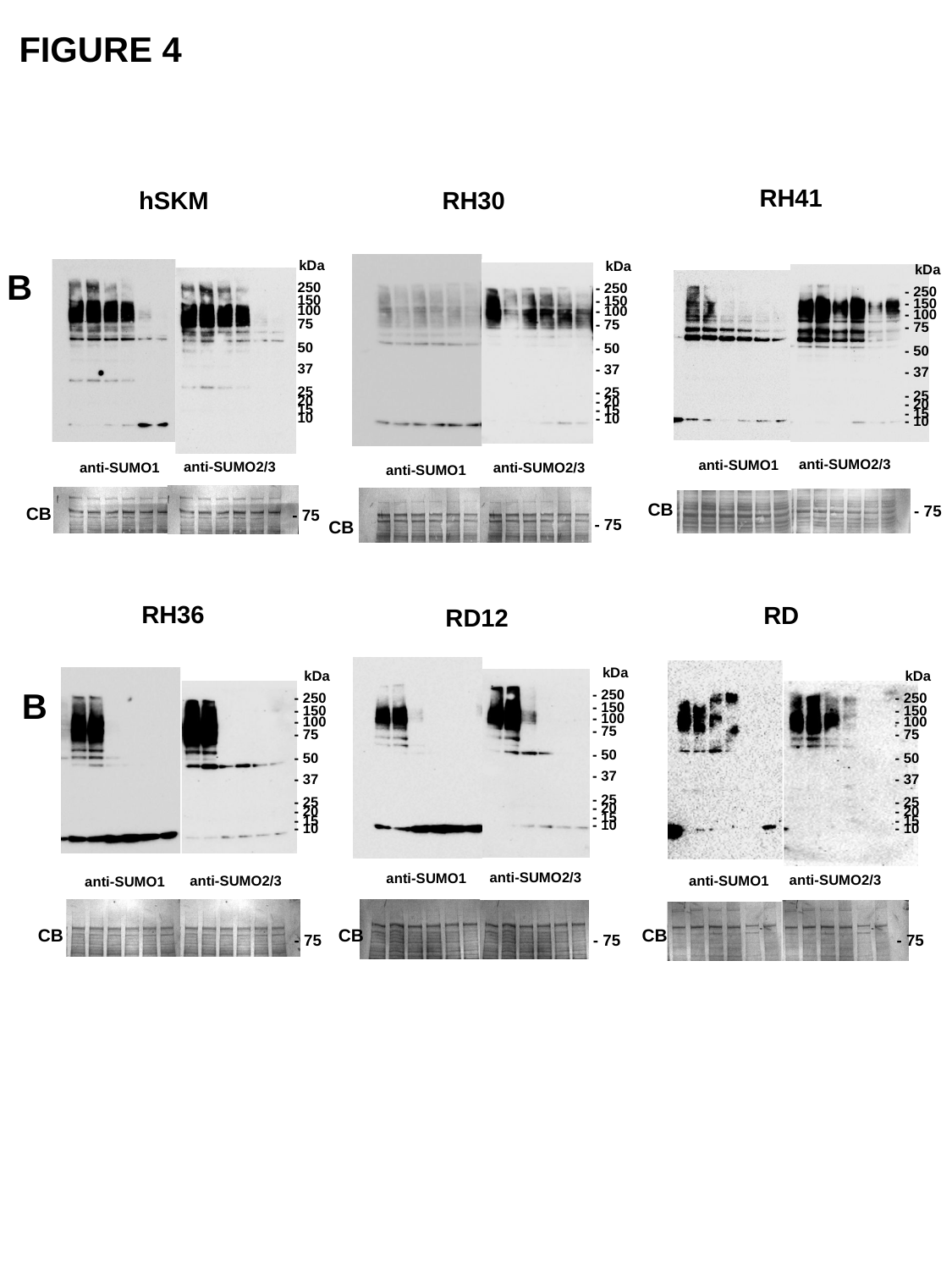

FIGURE 4
RH41
hSKM
RH30
kDa
- 250
- 150
- 100
- 75
- 50
- 37
- 25
- 20
- 15
- 10
kDa
- 250
- 150
- 100
- 75
- 50
- 37
- 25
- 20
- 15
- 10
kDa
- 250
- 150
- 100
- 75
- 50
- 37
- 25
- 20
- 15
- 10
B
anti-SUMO2/3
anti-SUMO1
anti-SUMO2/3
anti-SUMO1
anti-SUMO2/3
anti-SUMO1
CB
- 75
CB
- 75
- 75
CB
RH36
kDa
- 250
- 150
- 100
- 75
- 50
- 37
- 25
- 20
- 15
- 10
anti-SUMO2/3
anti-SUMO1
CB
- 75
RD
kDa
- 250
- 150
- 100
- 75
- 50
- 37
- 25
- 20
- 15
- 10
anti-SUMO2/3
anti-SUMO1
CB
- 75
RD12
kDa
- 250
- 150
- 100
- 75
- 50
- 37
- 25
- 20
- 15
- 10
B
anti-SUMO2/3
anti-SUMO1
CB
- 75

## Slide 5
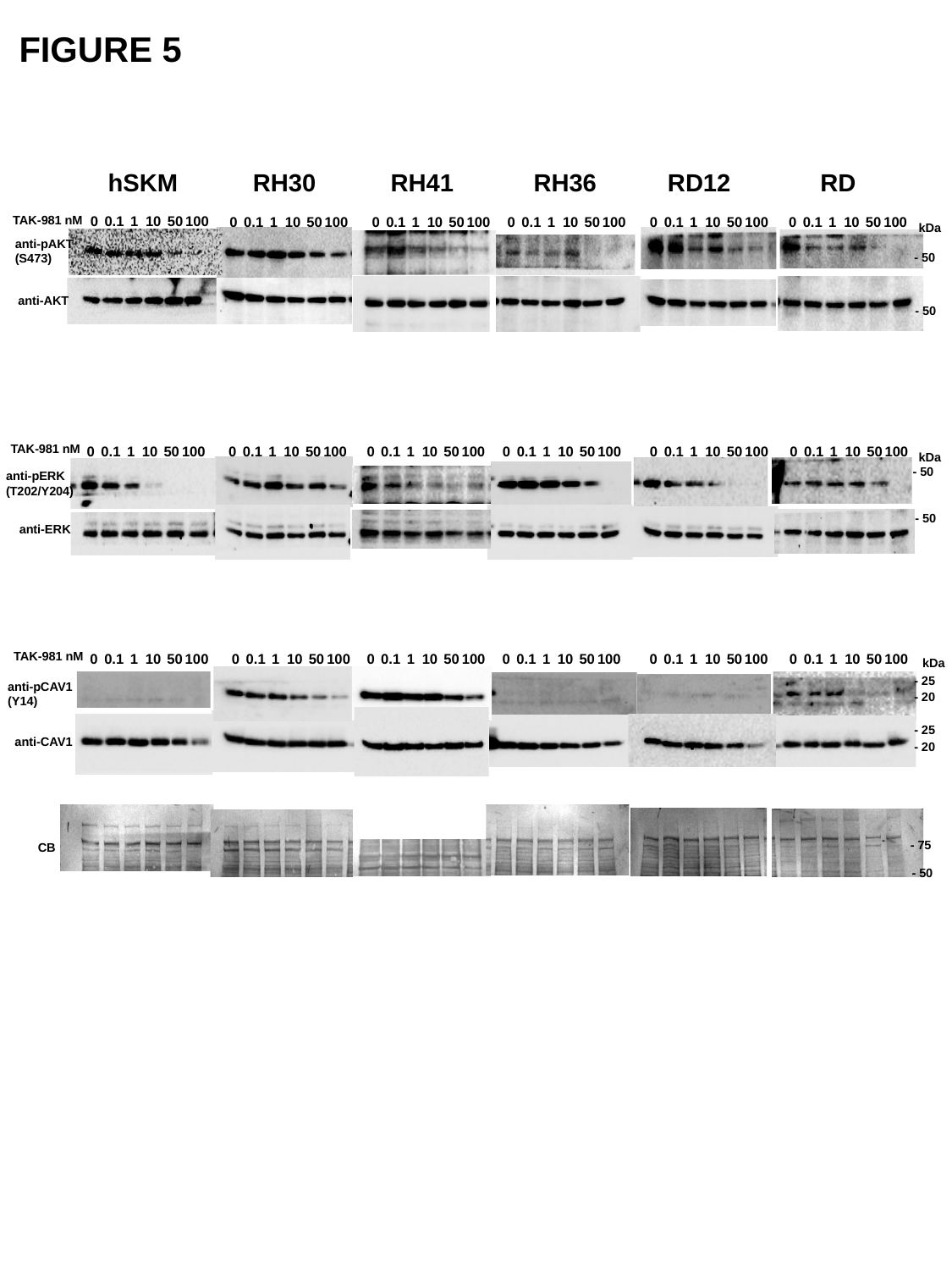

FIGURE 5
hSKM
RH30
RH41
RH36
RD12
RD
 TAK-981 nM
0
0.1
1
10
50
100
0
0.1
1
10
50
100
0
0.1
1
10
50
100
0
0.1
1
10
50
100
0
0.1
1
10
50
100
0
0.1
1
10
50
100
kDa
anti-pAKT (S473)
- 50
anti-AKT
- 50
 TAK-981 nM
0
0.1
1
10
50
100
0
0.1
1
10
50
100
0
0.1
1
10
50
100
0
0.1
1
10
50
100
0
0.1
1
10
50
100
0
0.1
1
10
50
100
kDa
- 50
anti-pERK (T202/Y204)
- 50
anti-ERK
 TAK-981 nM
0
0.1
1
10
50
100
0
0.1
1
10
50
100
0
0.1
1
10
50
100
0
0.1
1
10
50
100
0
0.1
1
10
50
100
0
0.1
1
10
50
100
kDa
- 25
anti-pCAV1 (Y14)
- 20
- 25
anti-CAV1
- 20
- 75
CB
- 50

## Slide 6
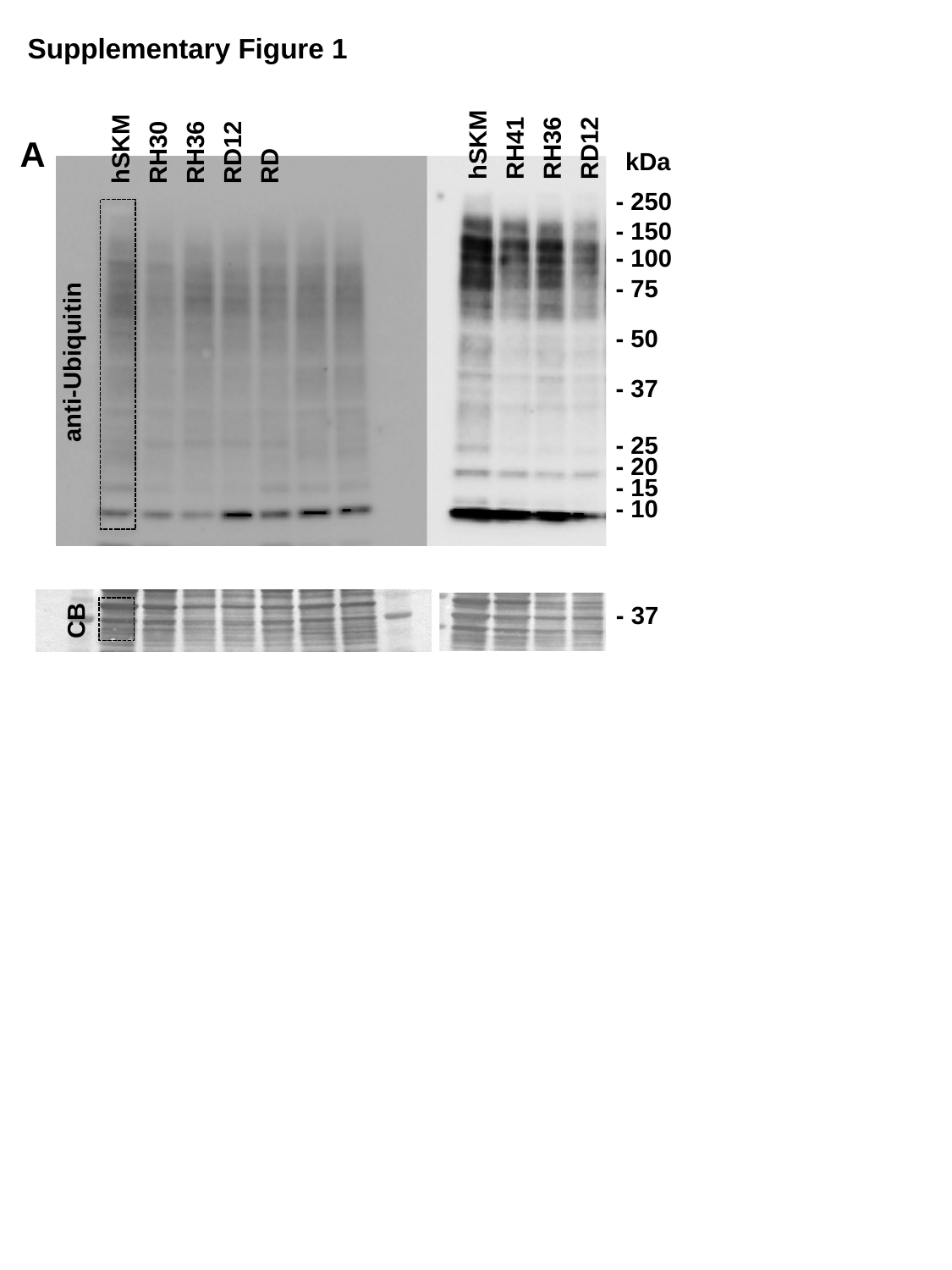

Supplementary Figure 1
RD
hSKM
RH41
RH36
RD12
hSKM
RH30
RH36
RD12
A
kDa
- 250
- 150
- 100
- 75
- 50
anti-Ubiquitin
- 37
- 25
- 20
- 15
- 10
- 37
CB

## Slide 7
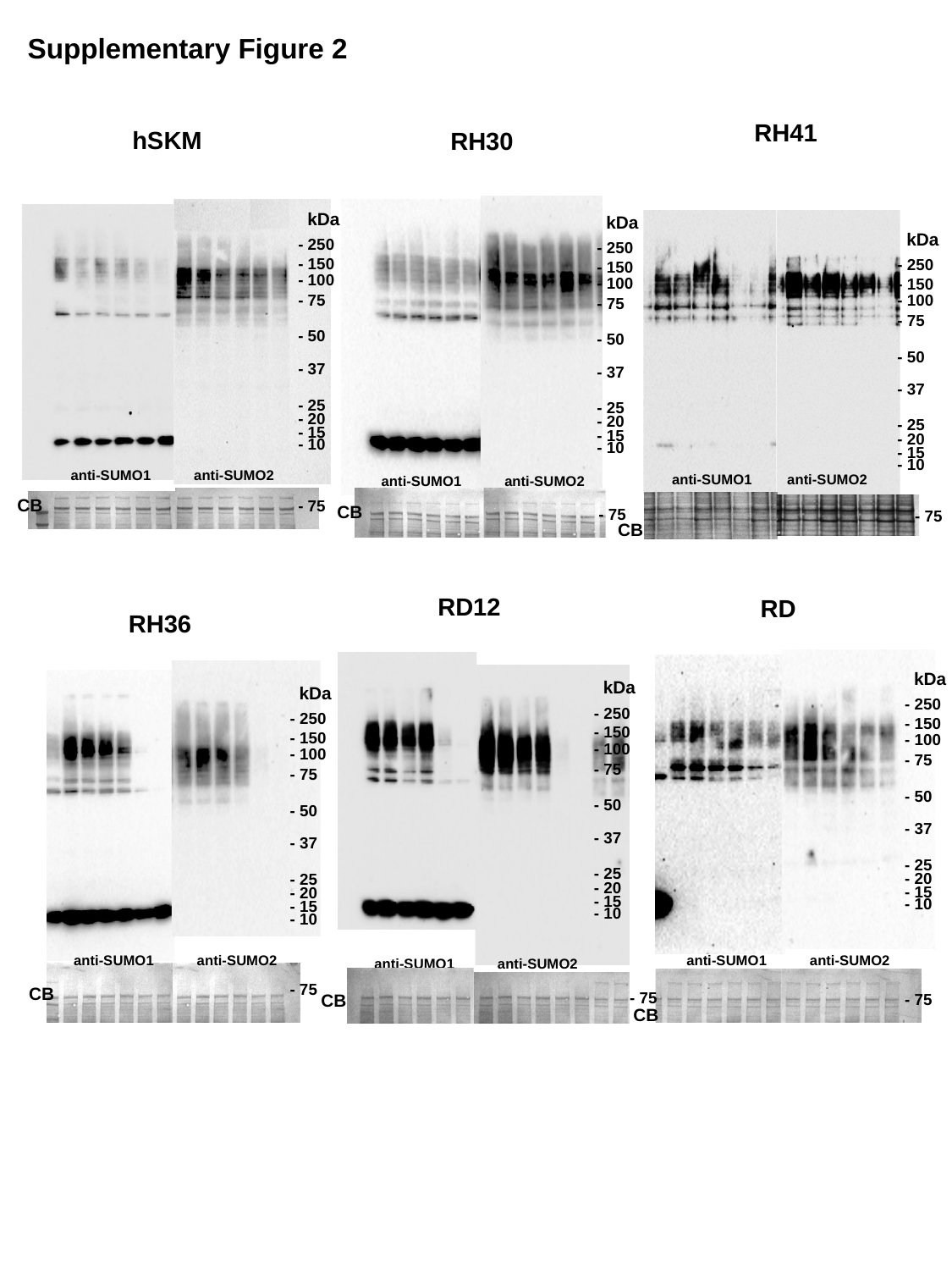

Supplementary Figure 2
RH41
hSKM
RH30
kDa
- 250
- 150
- 100
- 75
- 50
- 37
- 25
- 20
- 15
- 10
kDa
- 250
- 150
- 100
- 75
- 50
- 37
- 25
- 20
- 15
- 10
kDa
- 250
- 150
- 100
- 75
- 50
- 37
- 25
- 20
- 15
- 10
anti-SUMO1
anti-SUMO2
anti-SUMO1
anti-SUMO2
anti-SUMO1
anti-SUMO2
CB
- 75
CB
- 75
- 75
CB
RD12
RD
RH36
kDa
- 250
- 150
- 100
- 75
- 50
- 37
- 25
- 20
- 15
- 10
kDa
- 250
- 150
- 100
- 75
- 50
- 37
- 25
- 20
- 15
- 10
kDa
- 250
- 150
- 100
- 75
- 50
- 37
- 25
- 20
- 15
- 10
anti-SUMO1
anti-SUMO2
anti-SUMO1
anti-SUMO2
anti-SUMO1
anti-SUMO2
- 75
CB
- 75
CB
- 75
CB
