# Supplementary material for: Synthetic inhibition of the SUMO pathway by targeting the SAE1 component via TAK-981 compound impairs growth and chemosensitizes embryonal and alveolar rhabdomyosarcoma cell lines
Source: Mol Cell Biochem. 2025 Jun 23;480(10):5501–19. doi: 10.1007/s11010-025-05336-6 (PMC12515125; doi:10.1007/s11010-025-05336-6)
Supplement: Supplementary file 2 — Supplementary file2 (DOCX 17 KB) [file 11010_2025_5336_MOESM2_ESM.docx]

**Supplementary Figure 1.** Analysis of ubiquitin conjugates expression in RMS cell lines and control. (A) Immunoblot analysis of ubiquitin conjugates in RMS cell lines (RH30, RH41, RH36, RD12, RD) and human skeletal muscle (hSKM) used as control. Proteins were separated by SDS-PAGE and probed with anti-ubiquitin antibody. A Coomassie Blue (CB) stain of total protein is shown as a loading control. Molecular weight markers (kDa) are indicated on the right. (B) Quantification of signal intensity relative to the hSKM control. Data are presented as mean ± standard deviation from five independent experiments, except for RH41, n=3. The variation in band intensity is expressed as a percentage relative to hSKM. Statistics: **: p<0.005.

**Supplementary Figure 2.** Dose-dependent inhibition of SUMOylation by TAK-981 in RMS cell lines and human skeletal muscle (hSKM) control after 4 hours. Western blot and quantification of SUMO1 and SUMO2 conjugates in hSKM and RMS cell lines (RH30, RH41, RH36, RD12, RD) treated with varying concentrations of the SUMOylation inhibitor TAK-981 (0, 0.1, 1, 10, 50, 100 nM) for 4 hours. Blots were probed with anti-SUMO1 and anti-SUMO2 antibodies to detect SUMO-conjugated proteins. Coomassie Blue (CB) staining is provided as a loading control. Molecular weight markers (kDa) are indicated on the right. Top panels: Quantification of SUMO conjugate intensity in hSKM and RMS cell lines following TAK-981 treatment. Band intensity is expressed as a percentage relative to untreated control (0 nM). Data are shown as mean ± standard deviation from three independent experiments. Statistisc: *: p<0.05; **: p<0.005; ***: p<0,001. Bottom panels: Representative immunoblots showing the dose-dependent reduction of SUMO1 and SUMO2 conjugates across the cell lines. Molecular weight markers (kDa) are indicated on the right.

**Supplementary Figure 3.** Effect of TAK-981 on cell migration in RMS cell lines and human skeletal muscle (hSKM) control. (A) Wound healing (WH) quantification assay of the cell migration ability of hSKM and RMS cell lines (RH30, RH41, RH36, RD12, RD) following treatment with increasing concentrations of TAK-981 (0, 0.1, 1, 10, 50, 100 nM) for 8 hours (light gray columns) and 24 hours (dark gray columns). The percentage of the repaired area was calculated relative to the initial wound area at 0 hours. Data represent mean ± standard deviation from six independent experiments. Statistic: *: p<0.05; **: p<0.005; ***: p<0,001. (B) IC50 values of TAK-981. RMS and hSKM lines were treated with doses of TAK-981 ranging from 0.1 to 100 nM for 48 hours. The black dotted line corresponds to the 50% inhibition. The table shown the IC50 values of TAK-981 calculated using doses between 0.1 and 100 nM for 48 hours for each cell line reported.

**Supplementary Figure 4.** RH30, RH41, RH36, RD12, and RD were treated with chemotherapeutic agents Actinomycin D, (ActD), or Doxorubicin, (DOX) in combination with TAK-981. (A) Bar graphs shown cell proliferation at 24 hours and 48 hours under different treatment conditions: untreated (UT), TAK-981 (1 nM, 10 nM), ActD (0.5 nM), DOX (0.5 µM), and combination treatments. Each bar represents mean ± standard deviation from n=3 replicates. Statistics: *: p<0.05; **: p<0.005; ***: p<0,001, (A).
